# Recommendations for use of personal protective equipment (PPE) in surgical procedures during the SARS-Cov pandemic

**DOI:** 10.1590/1677-5449.200044

**Published:** 2021-06-16

**Authors:** Ana Alyra Garcia Carvalho, Ana Laura e Silva Aidar, Brena Costa dos Santos, Danielle Akemi Bergara Kuramoto, Mariana Raffo Pereda, Rebeca Mangabeira Correia, Luis Carlos Uta Nakano, Jorge Eduardo Amorim

**Affiliations:** 1 Universidade Federal de São Paulo – UNIFESP, Escola Paulista de Medicina – EPM, Departamento de Cirurgia Vascular e Endovascular, São Paulo, SP, Brasil.

**Keywords:** personal protective equipment, surgical procedures, coronavirus, COVID, SARS-Cov

## Abstract

Since the Coronavirus Disease 2019 was classified as a pandemic by the World Health Organization in 2019, many measures have been proposed to reduce the risks and the chances of contamination by the new coronavirus. In this context, wearing personal protective equipment is very important, especially in hospital environments and situations involving healthcare, since the degree of exposure is notably higher among the subgroup of healthcare professionals. The aim of this article is to propose a roadmap for the sequence of personal protective equipment use for surgical procedures during the coronavirus pandemic. The recommendations were based on Brazil’s public health policy and World Health Organization guidelines. Five roadmaps for PPE sequences are presented for the most commonly performed procedures: fitting central venous catheters; fitting catheters requiring radioscopy; open surgeries; diagnostic and therapeutic angiography, and dressings.

## INTRODUCTION

Since coronavirus 2019 disease (COVID-19) was classified as a pandemic by the World Health Organization (WHO), there has been much debate on the definitions of suspected cases and confirmed cases and the recommendations for use of the correct personal protective equipment (PPE) in different health care provision scenarios. This degree of concern is because of the extremely high numbers of cases reported daily in many parts of the world.

According to data from the WHO, up to August 18, 2020 there had been a total of 21,756,357 confirmed cases in 216 different regions globally, 771,635 (3.54%) of which were fatal.^1^ In Brazil, up to August 16, 2020, 3,407,354 COVID-19 cases had been confirmed, the majority of them concentrated in the country’s Southeast region (34.94%), where the state of São Paulo accounted for 59.75% of all confirmed cases in the Southeast.^2^

It is therefore extremely important to define suspected and confirmed cases of infection by the novel SARS-Cov in the current context, because doing so helps to define correct use of PPE and consequently to prevent the disease. According to the WHO recommendations,^3^^,^^4^ patients should be defined as suspect cases who have:

- Acute respiratory failure (combined with fever and at least one other symptom of respiratory tract infection, such as fever or dyspnea) and history of recent travel to or residence in an area in which community transmission of the disease has been confirmed during the 14 days prior to onset of the first symptoms;
- Acute respiratory failure and history of close contact during the last 14 days with a suspected or confirmed case of novel SARS-Cov infection.
- Acute respiratory failure, in the absence of any other diagnosis that explains the clinical presentation.

Confirmed cases require laboratory confirmation of novel SARS-Cov infection, irrespective of presence of symptoms.^3^ For cases in which laboratory confirmation is inconclusive or, despite a high degree of clinical suspicion patients cannot be tested in a laboratory for confirmation of infection, there is a third classification of probable case of infection.^3^

Transmission of novel SARS-Cov between humans was described for the first time in China and it is believed that it can be caused both by direct contact with respiratory droplets from sick and symptomatic patients^5^ and by indirect contact with infected people via contaminated hands, objects, or surfaces.^6^ Although transmission by asymptomatic individuals is considered controversial by the Brazilian Ministry of Health,^5^ evidence indicative of transmission by asymptomatic individuals has been from Germany, according to a case report published in the New England Journal of Medicine.^7^

Epidemiological surveillance and prevention are foregrounded in this context, since they aid in identification of suspect and/or confirmed cases and in implementation of preventative measures that reduce the risk of exposure and consequent contamination by the novel virus.

Since wearing PPE is the main preventative measure implemented in the different scenarios of health care provision, the objective of this article is to provide guidance on correct use of PPE by health professionals, with a particular focus on the surgical procedures conducted at our center. All of the information presented is in compliance with our institution’s Nosocomial Infection Control Committee, the Brazilian Ministry of Health, and the WHO.

## PPE

Personal protective equipment is defined as any device or product for personal use by a worker that is intended to protect them from risk situations that threaten their health and safety. The main PPE currently available, their respective applications, and precautions for their use are as follows:

- Surgical mask: should be worn to avoid contamination of the professional’s nose and mouth by respiratory droplets when working at distances of less than 1 meter from patients with suspected or confirmed novel SARS-Cov infection.^6^

The main precautions recommended when using are: cover both mouth and nose when wearing; do not touch the front of the mask; always use the ties or elastic bands for removal; always change masks that are soiled or humid; and do not reuse.^6^ Masks are recommended for all patients with suspected or confirmed novel SARS-Cov infection, and all health professionals who provide care less than 1 meter from suspected or confirmed cases.^6^

- PPF2/N95 or equivalent respiratory protection masks: indicated for procedures involving risk of generation of aerosols (intubation or tracheal aspiration, non-invasive ventilation, cardiopulmonary resuscitation), in both suspected and confirmed patients.^6^ Also recommended for professionals who work in surgical procedures requiring oral endotracheal intubation of suspected or confirmed cases, including surgeons performing procedures requiring general anesthesia.^6^^,^^8^

The specific recommendations and precautions vary depending on the model and manufacturer of the mask.^6^ However, in general, it is recommended that they should always be handled by the ties or elastic bands; the front of the mask should not be touched, because of the risk of contamination; and always dispose of masks that are humid, soiled, torn, or crushed.^9^

- Gloves: when the procedure to be performed on the patient demands aseptic technique, sterile gloves should be used.^6^ The main precautions to be taken when wearing and handling gloves are: perform hand hygiene after removing gloves; do not touch surfaces and materials unnecessarily when wearing gloves; and never reuse them;^6^
- Protective goggles or face shield: these are exclusive to each healthcare professional and should be worn whenever the professional is at risk of exposure to blood splashes, bodily secretions, excretions, etc.^6^ Goggles or face shield should always be sterilized: before and after wearing with alcohol 70% or another disinfectant recommended by the manufacturer;^6^
- Gown or apron: indicated to avoid contamination of the professional’s skin and clothes. Ideally, gowns or aprons should provide an effective antimicrobial barrier, have long sleeves, with knitted or elasticated cuffs, and back opening. Additionally, they should be removed and discarded as contaminated waste after performing the procedure and before leaving the patient’s room or the isolation area;^6^
- Cap: worn to protect professionals’ hair and head during procedures that could generate aerosols. They should be made from disposable material and removed after use. They should be discarded as contaminated waste.^6^

## GENERAL RECOMMENDATIONS FOR PREVENTION AND CONTROL OF THE NOVEL CORONAVIRUS

Measures for prevention and control of transmission of the novel SARS-Cov recommended by Brazil’s National Agency for Sanitary Vigilance (Agência Nacional de Vigilância Sanitária - ANVISA),^6^ in alignment with the WHO recommendations,^10^^,^^11^ stipulate the following precautions.

- Suspected or confirmed cases: wear surgical masks and use paper tissues (when there is coughing, sneezing, or nasal secretions); perform hand hygiene frequently with soap and water and/or alcohol 70%;
- Health professionals (in procedures that do not generate aerosols): wear protective goggles or face mask, surgical mask, apron, and scrub gloves and caps; perform hand hygiene;
- Health professionals (in procedures that do generate aerosols): wear protective goggles or face mask, PPF2/N95 or equivalent respiratory protection masks, apron, and scrub gloves and caps; perform hand hygiene.

In surgical settings, some further recommendations must also be followed that affect transporting the patient to the operating suite, induction of anesthesia, and the surgical operation itself. Transport should be performed unhindered and uninterrupted, ensuring priority use of elevators.^12^

With regard to precautions with anesthesia, ideally there should be an anteroom for healthcare professionals to prepare and an anesthesia induction room with negative pressure (if this is unavailable, it is recommended that air conditioning should be turned off for procedures that generate aerosols).^12^ The anesthesia cart should be restricted to individuals infected by the virus and should be kept in the anesthesia induction room, with medications needed available on a separate tray, so that no additional handling is needed.^12^ Materials for obtaining airways should be disposable.^12^ When the patient’s intensive care unit mechanical ventilator is exchanged for the operating suite ventilator, gas flow should be stopped and the oral endotracheal tube should be clamped.^12^ Finally, monitors and infusion pumps should be disinfected after the procedure is completed.^12^

The operating room should also be prepared according to certain principles, including: it should be distant from other operating rooms; it needs a good flow of circulation that is independent from that used for other patients; should have a negative pressure filter (preferably exclusively for this group of patients); and, during the pandemic, it is recommended that the same room is used for all surgical cases in infected patients, observing a minimum interval of 1 h between each procedure, to proceed with decontamination of the surgical suite with the institution’s standardized disinfectant.^12^^,^^13^

During the surgical operation itself, a dedicated circulating nurse should be available, in case any additional materials on the anesthesia cart are needed; workers who leave the room should remove their caps and gloves in the anteroom and perform hand hygiene.^12^ Additionally, all unused items and medications should be considered contaminated and disposed of and doors should be kept closed during the procedure.^12^

Still during surgery, instruments should be cleaned of blood or other secretions; the electric scalpel should be used at the lowest setting possible, to avoid smoke and dispersal of aerosols (taking care to avoid burn or cut injuries to team members);^6^ and, in suspected or confirmed cases de COVID-19, health professionals should always wear N95 or equivalent masks and sterilization of non-disposable material should be separated, as should waste disposal (which should be identified).^6^^,^^8^

**Figure 1:**
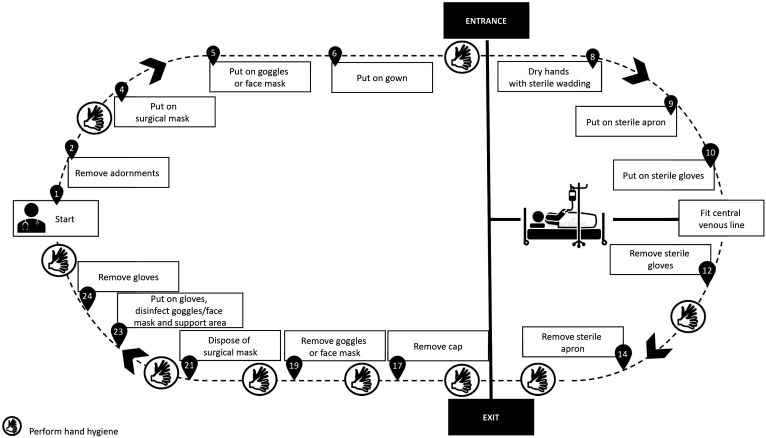
Fitting central venous catheters.

**Figure 2:**
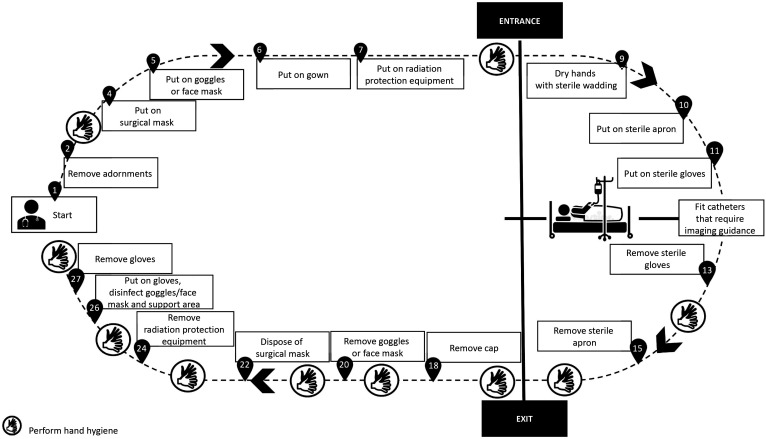
Fitting catheters that require imaging guidance.

**Figure 3:**
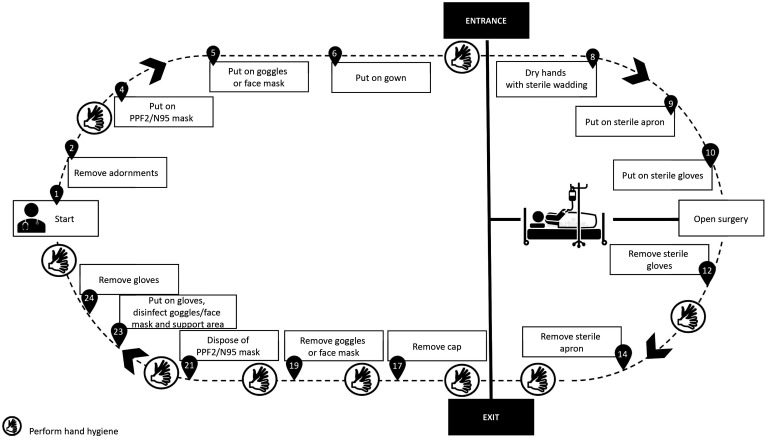
Open surgery.

**Figure 4:**
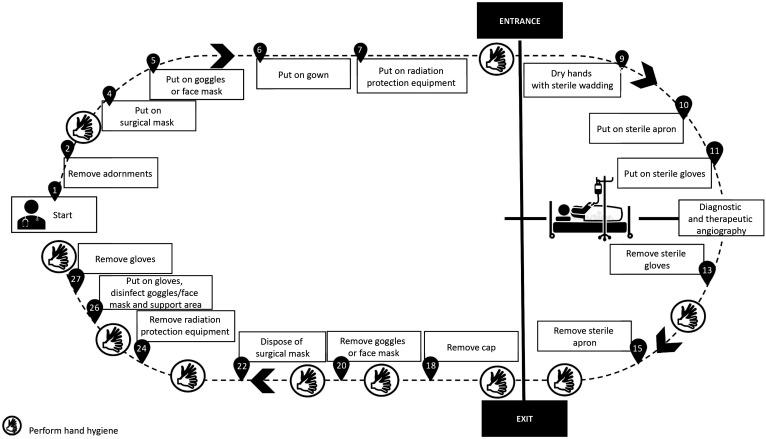
Diagnostic and therapeutic angiography.

**Figure 5:**
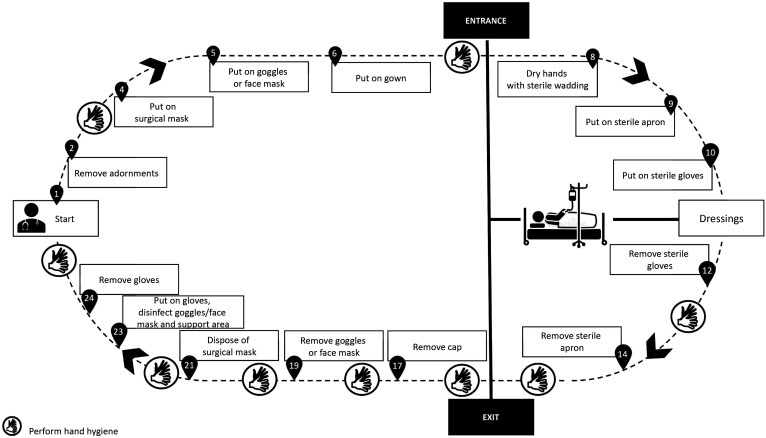
Dressings.

All of the precautions described above are intended to ensure a safer environment for health professionals and patients (suspected or confirmed cases of novel SARS-Cov infection) requiring surgical interventions. Below we suggest PPE sequences for the most common surgical procedures conducted routinely at our institution, namely: fitting central venous catheters (Figure 1); fitting catheters that require imaging guidance (Figure 2); open surgeries (Figure 3); diagnostic and therapeutic angiography (Figure 4); and dressings (Figure 5). All of the information presented is in compliance with our institution’s Nosocomial Infection Control Committee.

## CONCLUSIONS

In view of the current global situation caused by novel SARS-Cov infection, this article was needed to support preventative measures in surgical settings. Based on Brazilian Ministry of Health and WHO recommendations, we propose a model of preventative measures for a range of scenarios, primarily focused on surgical procedures.

The model proposed here reflects the situation in our department, but it applies the general principles of prevention and can be adapted for a range of other departments. In the context of the current pandemic, prevention is as important as treatment.
