# Supplementary material for: Recommendations for use of personal protective equipment (PPE) in surgical procedures during the SARS-Cov pandemic
Source: J Vasc Bras. 2021 Jun 16;20:e20200044. doi: 10.1590/1677-5449.200044 (PMC8218829; doi:10.1590/1677-5449.200044)
Supplement: Translation

# Translation: Recomendações de uso de equipamentos de proteção individual (EPIs) em procedimentos cirúrgicos durante a pandemia de SARS-Cov

**Authors:** Ana Alyra Garcia Carvalho, Ana Laura e Silva Aidar, Brena Costa dos Santos, Danielle Akemi Bergara Kuramoto, Mariana Raffo Pereda, Rebeca Mangabeira Correia, Luis Carlos Uta Nakano, Jorge Eduardo Amorim

**Affiliations:** 1 Universidade Federal de São Paulo – UNIFESP, Escola Paulista de Medicina – EPM, Departamento de Cirurgia Vascular e Endovascular, São Paulo, SP, Brasil.

**Keywords:** equipamentos de proteção individual, procedimentos cirúrgicos, coronavirus, COVID, SARS-Cov

## Abstract

Desde a classificação da doença por coronavírus 2019 (COVID-19) como pandemia pela Organização Mundial de Saúde (OMS), muitas medidas foram propostas com o intuito de diminuir os riscos e a chance de contaminação pelo novo coronavírus. Nesse contexto, o uso de equipamentos de proteção individual (EPIs) é de suma importância, especialmente em ambientes hospitalares e em situações que envolvem o cuidado em saúde, visto que o grau de exposição é notadamente maior no subgrupo de profissionais de saúde. Este artigo tem como objetivo propor um roteiro de uso de EPIs para procedimentos cirúrgicos. As recomendações sugeridas são estruturadas com base nas diretrizes do Ministério da Saúde e da OMS, e cinco roteiros de uso de EPIs foram sugeridos para os procedimentos mais comumente realizados: passagem de acesso venoso central; passagem de cateteres que necessitem de escopia; cirurgias abertas; angiografia diagnóstica e terapêutica; e curativos.

## INTRODUÇÃO

Desde a classificação da doença por coronavírus 2019 (COVID-19) como pandemia pela Organização Mundial de Saúde (OMS), há muita discussão a respeito das definições de casos suspeitos e de casos confirmados e quais as recomendações de uso de equipamentos de segurança individual adequados às diferentes situações referentes aos cuidados em saúde. Toda essa preocupação se deve à quantidade expressiva de casos reportados diariamente em diversos locais do mundo.

Segundo dados da OMS, até o dia 18 de agosto de 2020 houve um total de 21.756.357 casos confirmados em 216 regiões diferentes do mundo, dos quais 771.635 (3,54%) foram a óbito^1^. No Brasil, até o dia 16 de agosto de 2020 foram confirmados 3.407.354 casos de COVID-19, a maior parte deles concentrados na Região Sudeste (34,94%), sendo o estado de São Paulo responsável por 59,75% de todos os casos confirmados da Região Sudeste^2^.

Portanto, definir casos suspeitos e confirmados de infecção pelo novo SARS-Cov é de extrema importância no contexto atual, uma vez que auxilia no correto uso de equipamentos de proteção individual (EPIs) e, por conseguinte, na prevenção da doença. De acordo com as recomendações da OMS^3^^,^^4^, definem-se como casos suspeitos pacientes com:

- Insuficiência respiratória aguda (associada a febre e ao menos mais um sintoma de infecção do trato respiratório, como febre ou dispneia) e relato de viagem recente, ou residência em áreas nas quais já se confirmou transmissão comunitária da doença nos últimos 14 dias que antecederam o aparecimento do primeiro sintoma;
- Insuficiência respiratória aguda e relato de contato próximo nos últimos 14 dias com caso suspeito ou confirmado de infecção pelo novo SARS-Cov;
- Insuficiência respiratória aguda, na ausência de outro diagnóstico que justifique o quadro clínico.

Para casos confirmados, é necessária comprovação laboratorial de infecção pelo novo SARS-Cov, independentemente da presença de sintomas^3^. Nos casos em que a comprovação laboratorial for inconclusiva, ou, apesar da alta suspeição clínica, os pacientes não puderem realizar o teste laboratorial para a confirmação da infecção, há uma terceira classificação em casos prováveis de infecção^3^.

A transmissão pelo novo SARS-Cov entre humanos foi descrita pela primeira vez na China, e acredita-se que se deva tanto ao contato direto com gotículas respiratórias oriundas de pacientes doentes e sintomáticos^5^ quanto ao contato indireto com pessoas infectadas por meio das mãos, de objetos ou de superfícies contaminadas^6^. Apesar de a transmissão por indivíduos assintomáticos ser considerada controversa pelo Ministério da Saúde^5^, há indícios de transmissão por indivíduos assintomáticos reportadas na Alemanha, conforme relato de caso publicado no *New England Journal of Medicine* 7.

Nesse contexto, a vigilância epidemiológica, assim como a prevenção, ganham destaque, na medida em que auxiliam na identificação de casos suspeitos e/ou confirmados e na implementação de medidas preventivas que diminuem o risco de exposição e a consequente contaminação pelo novo vírus.

Sendo os EPIs a principal medida preventiva implementada nas diferentes áreas de atuação do cuidado em saúde, este artigo tem como objetivo orientar o uso adequado de EPIs pelos profissionais de saúde, dando especial enfoque para os procedimentos cirúrgicos realizados em nosso serviço. Todas as informações seguem as orientações da Comissão de Controle de Infecções Hospitalares do nosso serviço, em acordo com o Ministério da Saúde e a OMS.

## EPIS

Entende-se por EPI todo dispositivo ou produto de uso pessoal utilizado pelo trabalhador com o intuito de protegê-lo de situações de risco que ameaçam a sua saúde e segurança. Os principais EPIs disponíveis atualmente, e suas respectivas indicações e cuidados durante o uso, são:

- Máscara cirúrgica: devem ser utilizadas para evitar a contaminação do nariz e boca do profissional por gotículas respiratórias, quando este atuar a uma distância inferior a 1 metro do paciente suspeito ou confirmado de infecção pelo novo SARS-Cov^6^.

Os principais cuidados recomendados durante o uso são: cobrir a boca e o nariz ao vestir; não tocar na parte da frente da máscara; utilizar sempre as tiras laterais para remoção; trocar sempre que a máscara estiver suja ou úmida; e não reutilizar^6^. É recomendada para todos os pacientes suspeitos ou confirmados de infecção pelo novo SARS-Cov, e para todos os profissionais de saúde que prestem assistência a menos de 1 metro para casos suspeitos ou confirmados^6^.

- Máscara de proteção respiratória PPF2/N95 ou equivalente: indicada para procedimentos com risco de geração de aerossóis (intubação ou aspiração traqueal, ventilação não invasiva, ressuscitação cardiopulmonar), tanto em pacientes suspeitos quanto em pacientes confirmados^6^. Recomendada também para profissionais que atuem em procedimentos cirúrgicos que exijam intubação orotraqueal de caso suspeito ou confirmado, incluindo cirurgiões em procedimentos nos quais é necessária anestesia geral^6^^,^^8^.

As recomendações e os cuidados específicos de uso variam de acordo com o modelo e o fabricante da máscara^6^. Porém, de forma geral, recomenda-se sempre manuseá-la pelas tiras laterais; não tocar na frente da máscara, pelo risco de contaminação; e descartar a máscara sempre que estiver úmida, suja, rasgada ou amassada^9^.

- Luvas: quando o procedimento a ser realizado no paciente exigir técnica asséptica, devem ser utilizadas luvas estéreis^6^. Os principais cuidados durante o uso e a manipulação das luvas são: prosseguir a higiene de mãos após a retirada de luvas; não tocar desnecessariamente superfícies e materiais enquanto estiver com luvas; e jamais reutilizá-las^6^;
- Óculos de proteção ou protetores faciais (*face shield*): são exclusivos de cada profissional de saúde e devem ser utilizados quando houver risco de exposição do profissional a respingos de sangue, secreções corporais, excreções, etc^6^. Deve-se sempre higienizar os óculos ou protetores faciais antes e após o uso com álcool 70% ou outro desinfetante indicado pelo fabricante^6^;
- Capote ou avental: indicado para evitar a contaminação da pele e da roupa do profissional. Idealmente, os capotes ou aventais devem proporcionar barreira antimicrobiana efetiva, serem de mangas longas, com punhos de malha ou elástico, e apresentarem abertura posterior. Além disso, devem ser removidos e descartados como resíduos infectantes após a realização do procedimento e antes de sair do quarto do paciente ou da área de isolamento^6^;
- Gorro: utilizados para a proteção dos cabelos e da cabeça dos profissionais em procedimentos que podem gerar aerossóis. Devem ser de material descartável e ser removidos após o uso. Além disso, devem ser descartados como resíduos infectantes^6^.

## RECOMENDAÇÕES GERAIS NA PREVENÇÃO E CONTROLE DO NOVO CORONAVÍRUS

As medidas para a prevenção e o controle da disseminação pelo novo SARS-Cov recomendadas pela Agência Nacional de Vigilância Sanitária (ANVISA)^6^, em consonância com as recomendações da OMS^10^^,^^11^, estipulam os cuidados abaixo.

- Casos suspeitos ou confirmados: usar máscaras cirúrgicas e lenços de papel (quando apresentarem tosse, espirro ou secreção nasal); higienizar frequentemente as mãos com água e sabão e/ou álcool 70%;
- Profissionais de saúde (em procedimentos que não geram aerossóis): usar óculos de proteção ou protetor facial, máscara cirúrgica, avental, luvas de procedimento e gorro; higienizar as mãos;
- Profissionais de saúde (em procedimentos que geram aerossóis): usar óculos de proteção ou protetor facial, máscara N95/PPF2 ou equivalente, avental, luvas de procedimento e gorro; higienizar as mãos.

Em ambientes cirúrgicos, são necessárias ainda algumas recomendações adicionais, que impactam tanto no transporte do paciente até o centro cirúrgico quanto na indução anestésica e no ato cirúrgico em si. Quanto ao transporte, este deve ser feito de forma livre e sem interrupções, garantindo fluxo privilegiado aos elevadores^12^.

Quanto aos cuidados com anestesia, idealmente deve haver uma antesala para preparo do profissional de saúde e sala de indução anestésica com pressão negativa (na indisponibilidade desta, orienta-se desligar o ar condicionado durante a realização de procedimentos que geram aerossóis)^12^. O carrinho de anestesia deve ser específico para indivíduos infectados pelo virus e deve ser mantido na sala de indução anestésica, com a disponibilização dos medicamentos necessários em bandeja separada, de forma a não haver necessidade de manuseio adicional^12^. Os materiais para vias aéreas devem ser descartáveis^12^. Na troca do ventilador mecânico do paciente da unidade de terapia intensiva para o do centro cirúrgico, deve-se desligar o fluxo de gases e clampear o tubo orotraqueal com pinça^12^. Por fim, monitores e bombas de infusão devem ser desinfetados após o término do procedimento^12^.

A sala de cirurgia também deve ser preparada seguindo alguns preceitos, entre eles: deve ser distante das demais salas de cirurgia; necessita de bom fluxo de circulação independente dos demais pacientes; deve contar com filtro com pressão negativa (de preferência, específica para esse grupo de pacientes); e, durante a pandemia, recomenda-se utilizar a mesma sala para todos os casos cirúrgicos de pacientes infectados, mantendo um intervalo mínimo de 1 h entre cada procedimento, para proceder à descontaminação do centro cirúrgico com desinfetante padronizado da respectiva instituição^12^^,^^13^.

Durante o ato cirúrgico em si, um circulante específico deve estar disponível, caso seja necessário algum material adicional que esteja no carrinho da anestesia; ademais, funcionários que deixarem a sala devem retirar seus gorros e luvas na antessala e realizar higienização das mãos^12^. Além disso, todos os itens e medicamentos não utilizados devem ser considerados contaminados e descartados, e as portas devem permanecer fechadas durante o procedimento^12^.

Ainda durante a cirurgia, os instrumentos devem ser limpos de sangue ou de outras secreções; o bisturi deve ser utilizado em menor intensidade possível, para evitar fumaça e dispersão do aerossol (atentar também para lesões térmicas ou cortantes nas equipes)^6^; e, em caso suspeito ou confirmado de COVID-19, os profissionais de saúde devem sempre usar máscara N95 ou equivalente, e a esterilização de material permanente deve ser separada, incluindo o descarte de resíduos (os quais devem ser identificados)^6^^,^^8^.

Todos os cuidados acima descritos têm como objetivo proporcionar um ambiente seguro aos profissionais de saúde e ao paciente (caso suspeito ou confirmado de infecção pelo novo SARS-Cov) que necessite de intervenções cirúrgicas. Abaixo sugerimos um roteiro de uso de EPIs para os principais procedimentos cirúrgicos realizados rotineiramente em nosso serviço, respectivamente: passagem de acesso venoso central (Figura 1); passagem de cateteres que necessitem de escopia (Figura 2); cirurgias abertas (Figura 3); angiografia diagnóstica e terapêutica (Figura 4); e curativos (Figura 5). Todas as informações estão de acordo com as recomendações da Comissão de Controle de Infecção Hospitalar no nosso serviço.

**Figura 1:** Passagem de acesso venoso central.

**Figura 2:** Passagem de cateteres que necessitem de escopia.

**Figura 3:** Cirurgias abertas.

**Figura 4:** Angiografia diagnóstica e terapêutica.

**Figura 5:** Curativos.

## CONCLUSÃO

Devido ao atual cenário mundial de infecção pelo novo SARS-Cov, este artigo se faz necessário para auxiliar em medidas preventivas no ambiente cirúrgico. Com base em recomendações do Ministério da Saúde e da OMS, propomos um modelo de medidas preventivas em diversos cenários, voltado principalmente para procedimentos cirúrgicos.

O modelo aqui proposto representa a realidade do nosso setor, mas segue princípios gerais de prevenção, de forma que pode ser adaptado a diversos outros serviços. No contexto atual da pandemia, prevenir é tão importante quanto tratar.
